# Adult hypertension referral pathway and therapeutic management: British and Irish Hypertension Society position statement

**DOI:** 10.1038/s41371-023-00882-2

**Published:** 2024-01-09

**Authors:** Philip Lewis, Jacob George, Vikas Kapil, Neil R. Poulter, Sarah Partridge, James Goodman, Luca Faconti, Terry McCormack, Ian B. Wilkinson

**Affiliations:** 1 Stockport NHS Foundation Trust, Stockport, SK2 7JE UK; 2 Division of Molecular & Clinical Medicine, School of Medicine, Ninewells Hospital & Medical School, University of Dundee, Dundee, DD1 9SY UK; 3 William Harvey Research Institute, Centre for Cardiovascular Medicine and Devices, Queen Mary University London, London, EC1M 6BQ UK; 4 Imperial Clinical Trials Unit, School of Public Health, Imperial College London, London, W12 7RH UK; 5 Department of Primary Care and Public Health, Brighton and Sussex Medical School, University of Sussex, Brighton, BN1 9PH UK; 6 Department of Clinical Pharmacology and Therapeutics, Cambridge University Hospitals NHS Foundation Trust, Cambridge, CB2 0QQ UK; 7 King’s College London British Heart Foundation Centre, Department of Clinical Pharmacology, 4th Floor, North Wing, St. Thomas’ Hospital, Westminster Bridge, London, SE17EH UK; 8 Institute of Clinical and Applied Health Research, Hull York Medical School, Hull, HU6 7RX UK; 9 Division of Experimental Medicine, University of Cambridge, Addenbrooke’s Hospital, Cambridge, CB2 0QQ UK

**Keywords:** Hypertension, Diagnosis

## Abstract

In the UK, most adults with hypertension are managed in Primary Care. Referrals to Secondary Care Hypertension Specialists are targeted to patients in whom further investigations are likely to change management decisions. In this position statement the British and Irish Hypertension Society provide clinicians with a framework for referring patients to Hypertension Specialists. Additional therapeutic advice is provided to optimise patient management whilst awaiting specialist review. Our aim is to ensure that referral criteria to Hypertension Specialists are consistent across the UK and Ireland to ensure equitable access for all patients.

## Introduction

In the UK, most adults with hypertension are managed in Primary Care. National and international guidelines advise that adult referrals to Secondary Care Hypertension Specialists are targeted at patients in whom further investigations are likely to change management decisions [1–6]. Referrals are recommended when patients have raised blood pressure with life threatening target-organ damage (i.e. emergency/same day referrals); or when patients have, for example, suspected secondary hypertension, resistant hypertension, or complex polypharmacy (i.e. routine referrals) [1–6].

**Fig. 1:**
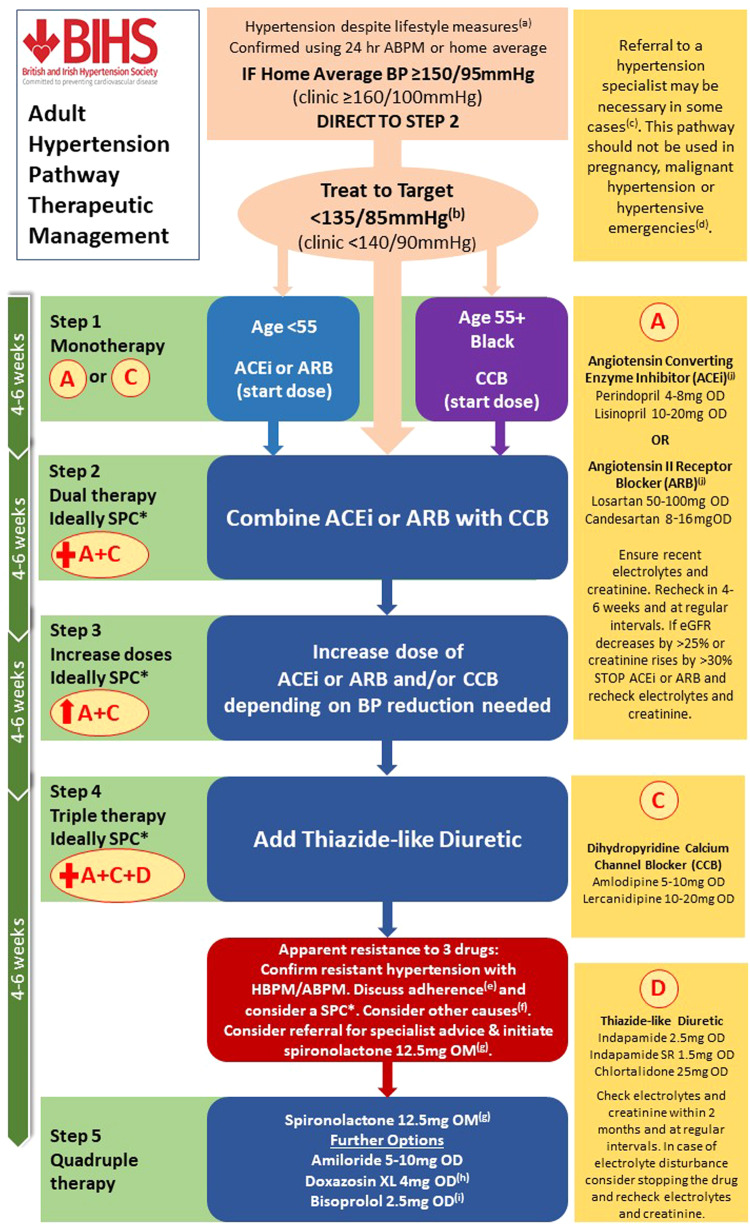
BIHS adult hypertension pathway therapeutic management. ABPM ambulatory blood pressure monitoring, ACEi angiotensin converting enzyme inhibitor, ARB angiotensin II receptor blocker, CCB calcium channel blocker, eGFR estimated glomerular filtration rate, HBPM home blood pressure monitoring, SPC single pill combination. *The availability of SPCs is currently limited in the UK and the only triple-component combination contains amlodipine, hydrochlorothiazide and olmesartan. Hydrochlorothiazide has been linked with an increased risk of skin cancer [9] and MHRA recommendations should be followed [10]. **a** Consider a trial of lifestyle optimisation for 3 months if BP is borderline elevated, especially where there are modifiable lifestyle risk factors including obesity, excess salt or excess alcohol intake. **b** Check for postural hypotension in those with frailty, aged >80 years, multi-morbidity, type 2 diabetes mellitus, Parkinson’s disease, or symptoms. In individuals with postural hypotension treat to a standing BP target. **c** See Table 2 for the criteria for routine referrals to a Hypertension Specialist. **d** See Table 1 for the criteria for emergency/same day referrals to a Hypertension Specialist. **e** Consider pre-payment certificates, dosette boxes, alarms or electronic reminders. **f** Encourage lifestyle modifications, including optimising body weight, salt and alcohol intake. Promote smoking cessation to reduce total cardiovascular risk. Re-review other drugs/supplements including: concomitant prescription of combined oral contraceptive pill or implant, hormone substitutes, steroids, NSAIDs, VEGF inhibitors, tyrosine kinase inhibitors (TKIs), tricyclic antidepressants, SSNRIs, dexamphetamine, methylphenidate, herbal supplements, illicit substances and liquorice. Consider co-existing medical conditions (e.g. sleep apnoea, aortic coarctation, chronic kidney disease). **g** To be avoided in patients with hyperkalaemia or at increased risk of developing hyperkalaemia. May be useful if hypokalaemia or heart failure. Can titrate to 25 or 50 mg. Check electrolytes and creatinine at each titration and ensure potassium remains <5.5 mmol/l (or upper limit of normality according to local laboratories). If feminising effect e.g. gynaecomastia, change to eplerenone at twice the dose, or amiloride. **h** May cause postural hypotension especially in the frail and older persons (ideally avoid), and in those with multiple co-morbidities. May cause stress incontinence in ~15% women. If a male is on another alpha blocker, e.g. Tamsulosin, then stop and use doxazosin for both hypertension and bladder outflow benefit. Doxazosin XL 4 mg OD or Doxazosin XL 8 mg OD have a smoother pharmacokinetic profile and reduce the incidence of postural hypotension. **i** Recommended in ischaemic heart disease. Avoid in asthma. May help with anxiety, although propranolol is likely to be more effective. **j** Consider halving the starting dose in those with heart failure (e.g. lisinopril 5 mg OD). Consider candesartan for dual BP control and migraine prophylaxis.

In this statement, the British and Irish Hypertension Society (BIHS) summarise their recommendations for adult emergency and routine referrals to Secondary Care Hypertension Specialists and highlight where this advice is supported by the National Institute for Health and Care Excellence (NICE) and/or International Societies (Tables 1 and 2) [1–6]. Table 3 summarises the ideal information to accompany referrals to facilitate communication between Primary and Secondary Care services. Where there are long waiting times to access routine Secondary Care hypertension services, the BIHS offer additional therapeutic advice to optimise patient management whilst awaiting specialist review (Fig. 1). Finally, the challenges facing referrers in identifying a Hypertension Specialist in the UK are discussed.

**Table 1:** BIHS criteria for emergency/same day referrals.

Clinical Situation	Supported by
Malignant/accelerated phase hypertension. Blood pressure ≥180/120 mmHg with retinal haemorrhages or papilloedema	NICE [1], ESC/ESH [4, 5]
Hypertensive crisis. Life threatening target-organ damage even in the context of mild or severe hypertension, including, but not limited to, acute aortic dissection, acute kidney injury, acute myocardial ischaemia, acute heart failure, acute stroke or phaeochromocytoma.	NICE [1], ESC/ESH [4, 5]
Pre-eclampsia and severe hypertension in pregnancy. Requires a multi-disciplinary team approach.	NICE [1, 3], ESC/ESH [4, 5]

*NICE* National Institute for Health and Care Excellence, *ESC* European Society of Cardiology, *ESH* European Society of Hypertension.

**Table 2:** BIHS criteria for routine referrals.

Clinical situation	Supported by
Aged under 40 years at diagnosis, irrespective of current age	NICE [1], ESC/ESH [4, 5]
Suspected secondary hypertension, including, but not limited to hyperaldosteronism (e.g. hypokalaemia); phaeochromocytoma (e.g. palpitations, headache, flushing, family history, history of neurofibromatosis); drug-induced hypertension (e.g. concomitant prescription of combined oral contraceptive pill or implant, hormone substitutes, steroids, NSAIDs, VEGF inhibitors, tyrosine kinase inhibitors (TKIs), tricyclic antidepressants, SSNRIs, dexamphetamine, methylphenidate). *Please note these are common examples but do not represent an exhaustive list of the secondary causes of hypertension*.	NICE [1], ESC/ESH [4, 5], ISH [6]
Hypertension in pregnancy (requires a multi-disciplinary team approach) AND women who remain hypertensive postpartum.	NICE [1, 3]
Resistant hypertension, defined as blood pressure uncontrolled on maximum tolerated doses of angiotensin converting enzyme inhibitor (ACEi) or angiotensin II receptor antagonist/blocker (ARB) + dihydropyridine calcium channel blocker (CCB) + thiazide-like diuretic (see Fig. 1).	NICE [1], ESC/ESH [4, 5], ISH [6]
Persistent symptomatic postural hypotension, despite medication adjustment (supine to standing after at least 1 minute, SBP falls by ≥20 mmHg and/or DBP falls by ≥10 mmHg).	NICE [1, 2]
Complex polypharmacy	ESC/ESH [4, 5]

*ESC* European Society of Cardiology, *ESH* European Society of Hypertension, *ISH* International Society of Hypertension, *NICE* National Institute for Health and Care Excellence, *NSAIDs* Nonsteroidal Anti-inflammatory Drugs, *SSNRI* selective serotonin noradrenaline reuptake inhibitor, *VEGF* vascular endothelial growth factor.

**Table 3:** Information to share with hypertension specialists.

History	Investigations (where possible)
• Reason for referral • Current medication • Previous intolerance to specific antihypertensive drugs with reasons • Relevant medical history and family history • Duration of hypertension / age at diagnosis	• Blood and urine test results • Ambulatory and/or home blood pressure monitoring results • ECG and/or echocardiography results • Imaging reports (e.g. CXR, renal ultrasound, CT or MRI)

*ECG* electrocardiogram, *CXR* chest x-ray, *CT* computed tomography, *MRI* magnetic resonance imaging.

## Adult referral criteria to a secondary care hypertension specialist

The clinical situations where the BIHS recommends emergency/same day referrals are outlined in Table 1.

The clinical situations where the BIHS recommends routine referrals are outlined in Table 2.

Prior to making routine referrals, clinicians should have:

1. Confirmed hypertension is present by either Ambulatory Blood Pressure Monitoring (ABPM) or Home Blood Pressure Monitoring (HBPM).
  AND if applicable,
2. Followed NICE guidelines NG136 on hypertension management [1], Fig. 1.
3. Assessed concordance with medication (preferably by urine testing, where available [7]).

## Information to share with hypertension specialists

To facilitate communication between referrers and Hypertension Specialists, the BIHS recommends that, where possible, the information in Table 3 is included with the referral. This avoids patients undergoing repeated testing unnecessarily, maximises the efficient use of resources and enables patients to start new, or modified, treatment regimens as soon as possible.

## Management advice for routine referrals awaiting hypertension specialist review

The BIHS acknowledges that current waiting times for routine NHS referrals may be considerable. To optimise hypertension treatment whilst patients await specialist review, clinicians may wish to consider the following steps:

- For newly diagnosed patients in whom secondary causes are suspected, or aged <40 years at diagnosis, or post-partum, a non renin-angiotensin-aldosterone system interfering drug is preferred (e.g. amlodipine or equivalent) as a temporary treatment while awaiting specialist review. This will facilitate interpretation of screening tests for the secondary causes of hypertension. Definitive long-term therapy should follow the guidance in Fig. 1.
- For those with established and uncontrolled hypertension, having tried multiple drug therapies, additional therapeutic options are summarised in Fig. 1. The choice of exemplar drugs within each class was based on the totality of evidence for each drug in reducing morbidity and mortality combined with duration of action. Long acting drugs are strongly preferred to minimise the impact of a missed dose and reduce blood pressure variability.
- Clinicians may also find patients are willing to revisit lifestyle modifications, including optimising weight, salt and alcohol intake and review their medication concordance whilst awaiting specialist advice. Re-assessing white coat hypertension by ABPM or HBPM can also be helpful [8].

## Selection of a hypertension specialist

It is important to refer patients to Hypertension Specialists who have appropriate training, experience and interest in hypertension management and who have access to specialist facilities to conduct relevant investigations. In the UK, there is currently no specialist registration for hypertension doctors and the only medical training curriculum that includes a specific module in hypertension is Clinical Pharmacology and Therapeutics. The BIHS is currently working on a system for accreditation and recognition of specialist hypertension services in the UK. In the interim, referrers can check if their local Hypertension Specialist is a member of the British and Irish Hypertension Society (Email: bihs@in-conference.org.uk), holds a European Hypertension Specialist certificate (https://www.eshonline.org/communities/hypertension-specialist/directory-of-specialists/) or works at a European Hypertension Centre of Excellence (https://www.eshonline.org/communities/excellence-centres/).
